# Extraction of pertechnetates from HNO_3_ solutions into ionic liquids

**DOI:** 10.1007/s10967-017-5362-3

**Published:** 2017-07-25

**Authors:** Maciej Chotkowski, Damian Połomski

**Affiliations:** 1 Faculty of Chemistry, University of Warsaw, Pasteura 1, 02-093 Warsaw, Poland; 2 Radiation Emergency Centre, National Atomic Energy Agency, Krucza 36, 00-522 Warsaw, Poland

**Keywords:** Pertechnetate, Extration, Ionic liquids

## Abstract

The extraction of pertechnetate ions from aquous solutions containing various concentrations of nitric acid into hydrophobic ionic liquids (ILs) has been examined at 25, 50 and 70 °C. The results show that the distribution ratio of Tc (D_Tc_) between both phases weakly depends on the temperature and HNO_3_ concentration when IL’s with relatively short aliphatic chains are used. The D_Tc_ obtained for all examined ILs, except methyltrioctylammonium bis(trifluoromethylsulfonyl)imide and 1-butyl-3-methylimidasolium hexafluorophosphate, are lower than 1.5. In the case of methyltrioctylammonium bis(trifluoromethylsulfonyl)imide a decrease of Tc concentration in aqueous solutions facilitates pertechnetate extraction into the organic phase.

## Introduction

Technetium, whose ^99^Tc isotope is one of the major long-lived products of ^235^U fission, is of particular interest in context of nuclear waste immobilization and reprocessing [1]. A high mobility of pertechnetate ions in aqueous media may lead to a long term contamination of environment with ^99^Tc. Although numerous works have been devoted to the extraction of technetium in liquid–liquid systems many aspects of ^99^Tc separation from spent nuclear fuel during reprocessing of the latter are nor fully understood and efficient removal of this isotope from other products of nuclear fission continues to pose challenging problems for nuclear industry. The distribution ratio of TcO_4_^–^, D_Tc_, has been determined for aqueous solutions in contact with organic systems, e.g. derivatives of ammonium chlorides in toluene [2], TBP in kerosine/dodecane [3–6], crown ether in m-xylene [7], tetraphenylphosphonium in chloroform/nitrobenzene [8], acetohydroxamic acid(AHA)/TBP [9] or tricaptylmethyl ammonium salt (Aliquat-336) [10]. Summary of the extraction properties of various extracting agents has been presented by Schwochau [11] and Spitsyn et al. [12]. Recent studies on this topic report an influence of presence of actinides on liquid–liquid Tc extraction [13–15].

Trace amounts of technetium can be extracted from aqueous basic solutions into an organic phase with the distribution ratio of up to 10^3^ [7]. Cocalia et al. [16] reported that D_Tc_ exceeds 700 in a system containing conc. K_3_PO_4_ and an ionic liquid (IL) phase and increases with increasing length of the aliphatic chain constituting the IL cation. These observations are in line with the results published by Schanker et al. [17]. They also reported an influence of the length of the aliphatic chain of the quaternary ammonium salts used as extracting agents on the extraction of pertechnetates in aqueous/chloroform systems. In the latter system the distribution ratio of Tc increases from 3.40 for tetrabutylammonium iodine to 9.89 for tetraheptylammonium iodine. This effect corresponds to direction of changes of other physical properties of organic derivatives of ammonium pertechnetates which are caused by increasing length of the aliphatic chain of the cation, i.e. a decrease in solubility in water and an increase of hydrophobic properties of the organic cation shown for tetra(C_n_H_2n+1_)ammonium pertechnetates series [18].

The solubility of ionic liquids in water is determined mainly by properties of their anions. As a general trend, ILs with [Tf_2_N^−^] ions are less soluble in water than ILs with [PF_6_^−^] anions. The respective solubility, which is represented as a molar fraction of the IL in water, increases from 10^−3.0–5.0^ for [A^+^][Tf_2_N^−^] to 10^−2.5^ for [A^+^][PF_6_^−^], [19].

Owing to their low solubility in water, the ionic liquids containing bis(trifluoromethylsulfonyl)imide or hexafluoro-phosphate anions represent an interesting alternative to classical extractants used in novel strategies for spent nuclear fuel reprocessing [20]. The main disadvantage of these extractans is their relatively low radiation stability [21] which limits their applications to reprocessing of the low level radioactive.

According to the literature [7], the extraction of pertechnetates in TcO_4_^−^_(aq)_/IL (A^+^B^−^) systems follows the *anion*-*exchange mechanism* presented by Eq. (1):

1 $${\text{TcO}}_{{4({\text{aq}})}}^{ - } + \, \left[ {{\text{A}}^{ + } } \right]\left[ {{\text{B}}^{ - } } \right]_{{ ( {\text{org)}}}} \rightleftharpoons \left[ {{\text{A}}^{ + } } \right]\left[ {{\text{TcO}}_{4}^{ - } } \right]_{{ ( {\text{org)}}}} + \, \left[ {{\text{B}}^{ - } } \right]_{{ ( {\text{aq)}}}}$$

This mechanism may change when crown-ethers (CE) are added to the IL [7]. Under such conditions the predominant mode of Tc transfer is described as the ion-pair production, according to Eq. (2):

2 $${\text{TcO}}_{{4 ( {\text{aq)}}}}^{ - } + {\text{ Na}}^{ + } + {\text{ CE}}^{ - }_{{({\text{org}})}} \rightleftharpoons \left[ {{\text{Na}} \cdot {\text{CE}}^{ + } } \right]\left[ {{\text{TcO}}_{4}^{ - } } \right]_{{({\text{org}})}}$$

The structure of the technetium complex extracted into the organic phase from aqueous HNO_3_ solutions was discussed also by El-Kot [4] who investigated tri-octylamine (TOA) and tri-n-butylphosphate (TBP) as extracting agents. It was proposed that pertechnetate anions must be neutralized by H^+^ in order to form neutral complexes with TOA or TBP:

3 $${\text{TcO}}_{{4 ( {\text{aq)}}}}^{ - } + {\text{ H}}^{ + } + {\text{ TOA}} \rightleftharpoons \left[ {{\text{HTcO}}_{4} \cdot {\text{TOA}}} \right]_{{ ( {\text{org)}}}}$$

4 $${\text{TcO}}_{{4 ( {\text{aq)}}}}^{ - } + {\text{ H}}^{ + } + \left( { 3 {\text{ or 4}}} \right){\text{ TBP}} \rightleftharpoons \left[ {{\text{HTcO}}_{ 4} \cdot \left( { 3 {\text{ or 4}}} \right){\text{ TBP}}} \right]_{{({\text{org}})}}$$

Depending on the HNO_3_ concentration, HTcO_4_ can coordinate three or four molecules of TBP. Noteworthy is the fact that for both TOA and TBP, El-Kot [4] observed a nonlinear relationship between the distribution ratio of Tc and the concentration of nitric acid. For low acid concentrations the D_Tc_ increases with the increase in the acidity of the aqueous phase. This effect is observed for HNO_3_ concentrations up to 0.1–0.2 mol dm^−3^ for TOA and up to 0.7–1 mol dm^−3^ for TBP. For higher concentrations of HNO_3_ the D_Tc_ decreases with the acid concentration for both extracting agents [4].

This manuscript reports results of preliminary studies on TcO_4_^−^ extraction with ionic liquids containing PF_6_^−^ and Tf_2_N^−^ anions. The experiments were carried out at various temperatures. The chemical properties of pertechnetates are very similar to that reported for perrhenates indicating that the topic of this manuscript is related also to various fields of industry and science where ReO_4_^−^ are used as reagents, e.g. hydrometallurgy.

## Experimental

Ionic liquids, [C_4_MIm][Tf_2_N], [S_222_][Tf_2_N], [P_666,14_][Tf_2_N], [N_4,111_][Tf_2_N], [N_1,888_][Tf_2_N], [C_2_Mpy] [Tf_2_N], [C_4_MIm][PF_6_] were obtained from IoLiTec and were used as received. The aqueous solutions were prepared using high purity distilled water (Millipore^®^, 18.2 Mohm cm) and high purity chemicals: potassium pertechnetate, K^99^TcO_4_ (Forschungszentrum Dresden-Rossendorf—Institute of Radiopharmacy) and HNO_3_ (POCh, Poland).

The extraction experiments were performed by contacting 1 ml of the aqueous solution containing pertechnetates with 1 ml of the ionic liquid. Temperature of the solutions studied was set at 25, 50 and 70 °C and was controlled using a Huber MPC E thermostat. After 30 min. of intense shaking the samples were centrifuged to facilitate phase separation, and the aliquots of both phases were assayed by a liquid scintillation counting method (Perkin Elmer Tri-Carb 2910TR) using Ultima Gold liquid scintillator cocktail. For each of the phases three samples of 20 microliters were collected and measured and the obtained results were averaged for each phase. The distribution ratio of Tc between organic and aqueous phase has been defined as the ratio of ^99^Tc activity in organic to the activity of ^99^Tc present in aqueous phase, D_Tc_ = A_Tc(org)_/A_Tc(aq)_.

The water content in the organic phases was determined by means of using Karl Fisher titration method (Metrohm, 716 DMS Titrino).

## Results and discussion

**Fig. 1:**
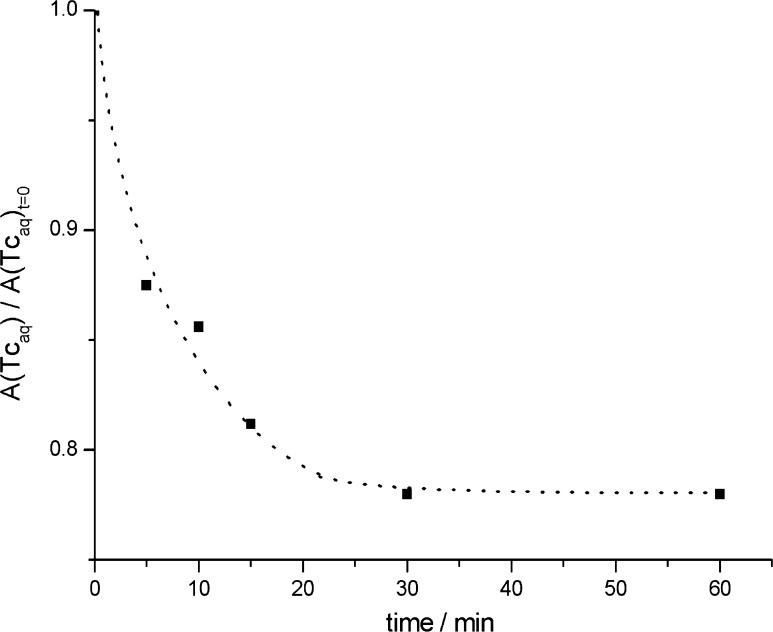
Activity, A_Tc_, of pertechnetates referred to initial activity of Tc, A(Tc_aq_)_t=0_ in aqueous phase as a function of contacting time of organic and aqueous phases

The optimum contacting time of organic and aqueous phases has been determined on the basis of experiments conducted in 0.06 mM TcO_4_^−^ (in 3 M HNO_3_)/[C_2_mpy][Tf_2_N] system. The degree of extraction of Tc from the aqueous into the organic phase as a function of the contacting time is presented in Fig. 1. It follows from Fig. 1 that the contacting time of 30 min is sufficient to obtain the maximum saturation of pertechnetates in IL.

The results of the studies on pertechnetates extraction from aqueous HNO_3_ solutions into the ILs studied are summarized in Table 1. Generally, the obtained results clearly show that for the solutions containing TcO_4_^−^ at a micromole level and for most of the ILs studied the distribution ratio of Tc is low and does not exceed unity. The only exceptions are [N_1,888_][Tf_2_N], [P_666,14_][Tf_2_N] and [C_4_MIm][PF_6_] for which the distribution ratio is greater than 1 but still lower than 20.

**Table 1:** Distribution ratios of ^99^Tc between the aqueous phase (pure water or containing nitric acid, with 0.06 mmol dm^−3^ of TcO_4_^−^) and the ionic liquid phase (uncertainty: ± 0.01 with the exception of [C_4_MIm][PF_6_] for which the uncertainties are presented separately)

Temp. (°C)	Water	Concentration of nitric acid (mol dm^−3^)						
		0.1	0.7	2	3	4	6	8
[N_4,111_][Tf_2_N]								
25	0.25	0.18	0.14	0.12	0.12	0.13	0.13	0.11
50	0.23	0.18	0.14	0.13	0.12	0.11	0.12	0.11
70	0.20	0.16	0.15	0.14	0.14	0.13	0.11	0.15
[N_1,888_][Tf_2_N]								
25	10.81	18.85	2.03	0.37	0.22	0.17	0.13	0.14
50	11.80	16.52	2.36	0.51	0.30	0.26	0.22	0.16
70	9.98	11.40	2.21	0.56	0.36	0.29	0.22	0.17
[P_666,14_][Tf_2_N]								
25	0.94	1.16	1.20	0.39	0.24	0.07	0.07	0.06
50	0.87	1.69	0.57	0.34	0.25	0.09	0.09	0.10
70	0.86	1.37	0.31	0.29	0.25	0.10	0.10	0.16
[S_222_][Tf_2_N]								
25	0.25	0.22	0.19	0.18	0.16	0.15	0.17	0.19
50	0.24	0.21	0.19	0.17	0.17	0.18	0.20	0.20
70	0.25	0.19	0.19	0.18	0.17	0.19	0.26	0.22
[C_2_Mpy][Tf_2_N]								
25	0.30	0.26	0.21	0.18	0.15	0.16	0.17	0.25
50	0.25	0.24	0.20	0.18	0.18	0.18	0.17	0.18
70	0.24	0.20	0.20	0.19	0.20	0.20	0.20	0.20
[C_4_MIm][Tf_2_N]								
25	0.56	0.43	0.32	0.19	0.18	0.16	0.16	0.16
50	0.50	0.45	0.30	0.22	0.21	0.17	0.16	0.09
70	0.46	0.39	0.28	0.24	0.18	0.16	0.16	0.09
[C_4_MIm][PF_6_]								
25	4.86 ± 0.11	4.14 ± 0.09	2.83 ± 0.06	1.57 ± 0.03	1.20 ± 0.03	0.98 ± 0.02	0.80 ± 0.02	*
50	4.57 ± 0.10	3.77 ± 0.08	2.56 ± 0.05	1.51 ± 0.03	1.20 ± 0.03	*	*	*
70	3.37 ± 0.07	3.11 ± 0.06	2.55 ± 0.05	2.03 ± 0.04	1.66 ± 0.03	*	*	*

* Disappearance of the boundary between the aqueous and organic phase

The concentration of nitric acid in the aqueous phase does not play a significant role in the extraction of pertechnetates from aqueous into the organic phase for the systems containing ionic liquids with cations with relatively short aliphatic chains, i.e. [N_4,111_][Tf_2_N], [S_222_][Tf_2_N], [C_2_Mpy][Tf_2_N] and [C_4_MIm][Tf_2_N]. Noteworthy is the fact that the stability of IL containing PF_6_^−^ strongly depends on the HNO_3_ concentration. In acidic solutions this anion undergoes a hydrolysis process with participation of hydrogen cations, according to Eq. (5):

5 $${\text{PF}}_{ 6}^{ - } + {\text{ 4H}}_{ 2} {\text{O }} + {\text{ H}}^{ + } \to {\text{H}}_{ 3} {\text{PO}}_{ 4} + {\text{ 6 HF}}$$

This process manifests itself by disappearance of the boundary between concentrated nitric acid (≥4 M HNO_3_) and the organic phase. Without a doubt, the reaction (5) takes place also in the systems with lower HNO_3_ concentration but is too slow as to be completed in the timescale of the experiment. This leads to higher uncertainties of D_Tc_ determined for [C_4_MIm][PF_6_] as compared to the other systems (Table 1).

Our results show a clear impact of HNO_3_ concentration on D_Tc_ only for the largest cations with the biggest size. In the case of [N_1,888_][Tf_2_N] the D_Tc_ decreases with the increase in the acid concentration.

The highest value of the distribution ratio of the pertechnetates measured for all ILs studied is equal to 18.85 and was obtained for [N_1,888_][Tf_2_N] in 0.1 M HNO_3_ at 25 °C. A comparison with typical extracting agents used for TcO_4_^−^ extraction shows that the highest D_Tc_ value obtained for the ILs studied in this work (18.85) is higher than D_Tc_ obtained for 30% TBP (D_Tc_ equal to unity) but is significantly lower than the value of 250 measured for TOA (0.1 M) by El Kot [4]. Billard et al. [22] reported that the solubility of HNO_3_ in ILs increases with increasing the acid concentration which indicates that the interaction between TcO_4_^−^ and HNO_3_ dissolved in the organic phase should be considered. This point may be discussed on the basis of the measured influence of HNO_3_ concentration on the extraction of TcO_4_^−^ anions. An increase in HNO_3_ concentration impedes transfer of the pertechnetates into the organic phase. This observation suggests that the formation of a neutral complex, e.g. [HTcO_4_][A^+^B^−^], can but probably does not play a significant role in transferring of pertechnetates into the hydrophobic phase. Moreover, at high concentrations of HNO_3_ the NO_3_^−^ anions may compete with pertechnetates in the extraction process.

The protonation of pertechnetate ions in acidic solutions results in existence of undissociated pertechnetic acid molecules. Various values of the HTcO_4_ acidity constant, log_10_K_c_, are reported in the literature with the range of −0.4–0.6 [23]. These undissociated and neutrally charged HTcO_4_ molecules can be directly transferred into the organic phase according to the following equations (Eqs. 6–7):

6 $${\text{TcO}}_{{4 ( {\text{aq)}}}}^{ - } + {\text{ H}}^{ + } \rightleftharpoons {\text{HTcO}}_{{ 4({\text{aq}})}}$$

7 $${\text{HTcO}}_{{4 ( {\text{aq)}}}} + \, \left[ {{\text{A}}^{ + } } \right]\left[ {{\text{B}}^{ - } } \right]_{{({\text{org}})}} \rightleftharpoons \left[ {{\text{A}}^{ + } {\text{B}}^{ - } \cdot {\text{HTcO}}_{ 4} } \right]_{{({\text{org}})}}$$

As it was mentioned earlier, the solubility of bis(trifluoromethylsulfonyl)imide salts with organic cations in the aqueous phase is low [19] but replacement of organic cations with potassium increases the solubility of such IL up to 10 g/dm^−3^ = 31 mmol dm^−3^ [24], which is the value sufficiently high as to transfer the organic anion into the aqueous phase. Thus, taking into account Eq. 1 one may suggest that the technetium complex in the organic phase could be also described as:

8 $${\text{TcO}}_{{4 ( {\text{aq)}}}}^{ - } + \, \left[ {{\text{A}}^{ + } } \right]\left[ {{\text{Tf}}_{ 2} {\text{N}}^{ - } } \right]_{{({\text{org}})}} \rightleftharpoons \left[ {{\text{A}}^{ + } } \right]\left[ {{\text{TcO}}_{4}^{ - } } \right]_{{({\text{org}})}} + \, \left[ {{\text{Tf}}_{ 2} {\text{N}}^{ - } } \right]_{{({\text{aq}})}}$$

When the aqueous and organic phases are brought into contact, a small amount of the water dissolves in the organic phase. The amount of such dissolved water was determined for all examined ILs after completing the extraction experiments with the aqueous phase not containing HNO_3_ (Table 2). The highest water content in the range of 10 000 ppm was determined for the ionic liquids containing cations with short aliphatic chains while for more hydrophobic ILs, i.e. [N_1,888_][Tf_2_N] or [P_666,14_][Tf_2_N], the water content was almost an order of magnitude lower. The water content obtained for [C_4_MIm][Tf_2_N] is in line with the literature data [19]. The results obtained for [C_4_MIm][Tf_2_N] and [C_4_MIm][PF_6_] indicate that the water content in the ionic liquid increases with the increase in solubility of the latter in water.

**Table 2:** Concentration of water in ionic liquids after completing the extraction of pertechnetates from a HNO_3_ free aqueous phase containing 0.06 mM KTcO_4_

Ionic liquid	H_2_O concentration (in ppm) after extraction of TcO_4_^−^
[N_4,111_][Tf_2_N]	14,037
[N_1,888_][Tf_2_N]	1991
[P_666,14_][Tf_2_N]	1978
[S_222_][Tf_2_N]	17,243
[C_2_Mpy][Tf_2_N]	14,632
[C_4_MIm][Tf_2_N]	13,552
[C_4_MIm][PF_6_]	23,680

A comparison of data presented in Tables 1 and 2 indicates that the concentration of the water in the investigated ILs (as measured after completing the extraction) does not play significant role in the transfer of pertechnetates between the aqueous and the organic phase. For the HNO_3_ free aqueous phase the highest value of D_Tc_ is equal to 10.81 (at 25 °C) and is observed for one of the driest ILs, [N_1,888_][Tf_2_N], while for the more hydrophilic [S_222_][Tf_2_N] one of the lowest D_Tc_ values (0.25) was obtained. For the second driest IL, [P_666,14_][Tf_2_N], the D_Tc_ is low again and equals 0.94.

Interesting results were obtained for ILs containing the same cation and different anions, i.e. [C_4_MIm][Tf_2_N] and [C_4_MIm][PF_6_]. A decrease of IL solubility in water due to replacement of [PF_6_^−^] with [Tf_2_N] leads to an increase in Tc distribution ratio. For instance, the D_Tc_ determined in 0.1 M HNO_3_/IL at 25 °C equals 4.14 for [C_4_MIm][PF_6_] and is approximately ten times higher than the value measured for [C_4_MIm][Tf_2_N] under the same experimental conditions.

**Fig. 2:**
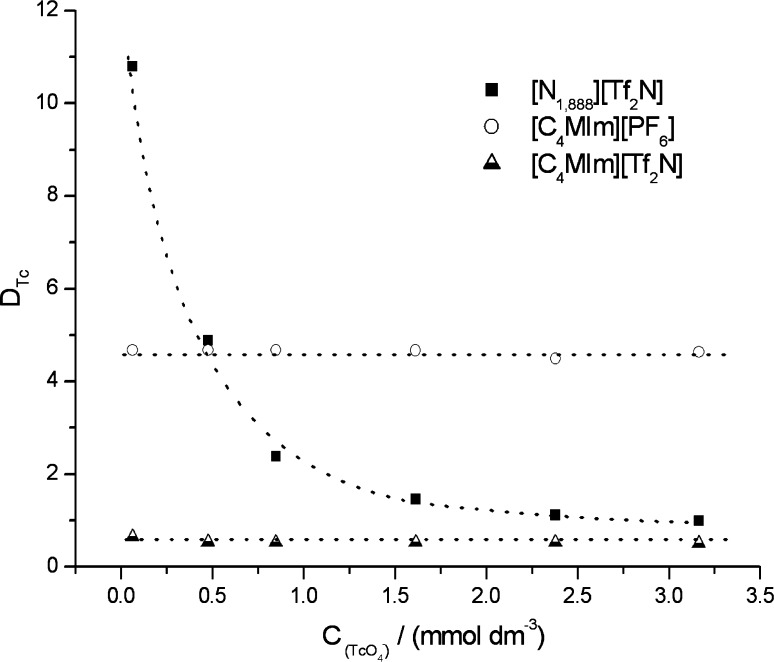
Distribution ratio (D_Tc_) of pertechnetates between aqueous (HNO_3_ free) and IL phases as a function of TcO_4_^−^ concentration

The influence of TcO_4_^−^ concentration on the extraction process was evaluated for the systems for which the highest ([N_1,888_][Tf_2_N], [C_4_MIm][PF_6_]) and the lowest ([C_4_MIm][Tf_2_N]) values of D_Tc_ were measured (Fig. 2). The influence of TcO_4_^−^ concentration on the extraction process is observed only for [N_1,888_][Tf_2_N], in this case the D_Tc_ significantly increases with the decreases in the pertechnetates concentration in the aqueous phase and for the technetium concentration at a micromole level the D_Tc_ values higher than 10 are obtained. On the other hand, the extraction properties of [C_4_MIm][PF_6_] and [C_4_MIm][Tf_2_N] are practically independent on the pertechnetates concentration.

**Fig. 3:**
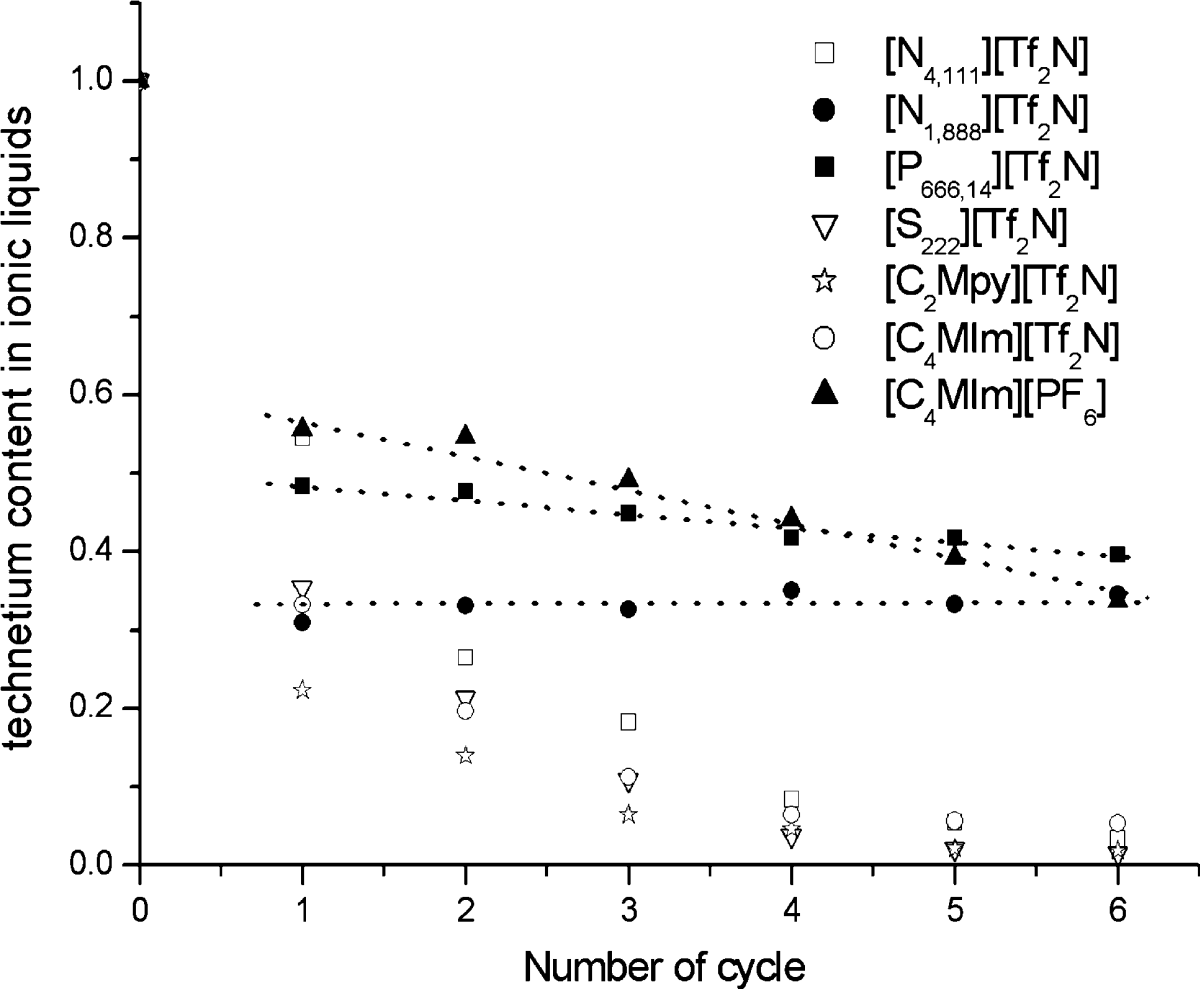
Yield of TcO_4_^−^ remained in IL after their removal with water (v/v: 1:1) as a function of number of back-extraction cycles. The concentration of TcO_4_^−^ is expressed in relation to their initial content in the specified IL

Repeated application of the same IL in the extraction process requires its purification by means of the back-extraction of Tc species into an aqueous phase. Usually, the removal of pertechnetates from the IL phase cannot be completed in a single cycle of the back-extraction process. Therefore, usually several consecutive back-extraction cycles are performed in order to obtain satisfying level of removal of TcO_4_^−^ from the ionic liquid. Figure 3 presents changes in concentration of pertechnetates remaining in the organic phase after specified number of the back-extraction cycles. The significant decrease in concentrations of Tc in organic phases are observed only for the first cycle of back-extraction, from 77 to 42% for [C_2_Mpy][Tf_2_N] and [C_4_MIm][Tf_2_N] respectively. If we assume that in organic phase are present not only [A^+^][TcO_4_^−^] but also undissociated HTcO_4_ molecules, the first contact of organic phase (saturated in technetium species) with water, results in effective transfer of HTcO_4_ into aqueous phase and there its dissociation to TcO_4_^−^ and H^+^ ions. For ILs which exhibit smallest affinity to pertechnetates, i.e. [C_2_Mpy][Tf_2_N], [S_222_][Tf_2_N], [N_4,111_][Tf_2_N], [C_4_MIm][Tf_2_N], only few purification steps are required for almost complete removal of TcO_4_^−^ from the organic phase. In the case of [N_1,888_][Tf_2_N] the pertechnetates are strongly bonded in the organic phase so that their back-extraction into the aqueous phase is practically insignificant.

This observation is in line with the results presented by Peretrukhin et al. [18] which show an extremely low solubility of tetra(C_n_H_2n+1_)ammonium pertechnetates salts with long aliphatic chain in water. For the other investigated ILs a slow decrease in the Tc concentration in the organic phase with the number of purification cycles is observed.

## Conclusions

The extraction properties of ionic liquids in respect to pertechnetates depend on the length of the aliphatic chain of the IL cation. The longer the aliphatic chain the higher the distribution ratio of pertechnetates between the aqueous and the organic phase. D_Tc_ higher than ten is observed only for [N_1,888_][Tf_2_N], the other ILs investigated in this work exhibit lower extent of the pertechnetates extraction. This factor increases with the decreases in Tc concentration in the aqueous phase. The presence of nitric acid in the aqueous phase leads to a decrease in the Tc transfer between the aqueous and the organic phases. Back-extraction of pertechnetates from the organic into the aqueous phase is efficient only for weakly extracting ILs.

## Abbreviations

[C_4_MIm][Tf_2_N]: 1-Butyl-3-methylimidasolium bis(trifluoromethylsulfonyl)imide
[S_222_][Tf_2_N]: Triethylsulfonium bis(trifluoromethylsulfonyl)imide
[P_666,14_][Tf_2_N]: Trihexyltetradecylphosphonium bis(trifluoromethylsulfonyl)imide
[N_4,111_][Tf_2_N]: Butyltrimethylammonium bis(trifluoromethylsulfonyl)imide
[N_1,888_][Tf_2_N]: Methyltrioctylammonium bis(trifluoromethylsulfonyl)imide
[C_2_Mpy][Tf_2_N]: 1-Ethyl-2-methylpyridinium bis(trifluoromethylsulfonyl)imide
[C_4_MIm][PF_6_]: 1-Butyl-3-methylimidasolium hexafluorophosphate
